# Positron emission tomography imaging of amyloid-beta plaque deposition: a decade of translation

**DOI:** 10.1186/1479-5876-10-S2-A31

**Published:** 2012-10-17

**Authors:** Julie C Price, Chester A Mathis, William E Klunk

**Affiliations:** 1 Departments of Radiology, University of Pittsburgh, Pittsburgh PA, 15213, USA; 2 Departments of Psychiatry, University of Pittsburgh, Pittsburgh PA, 15213, USA

## 

Positron emission tomography (PET) radiotracer developments over the past decade have enabled in vivo measurement of amyloid-beta (Aβ) plaque deposition, a key neuropathological hallmark of Alzheimer’s disease (AD). This presentation will provide an overview of the translational research path for one of the most widely used PET Aβ imaging agents, ^11^C-lableled Pittsburgh compound-B ([^11^C]PiB). Early preclinical ex-vivo characterization revealed the capacity of [^11^C]PiB to bind to fibrillar Aβ plaques [1]. Real-time *in vivo* multiphoton microscopy demonstrated that [^11^C]PiB labeled individual Aβ plaques in transgenic mouse models of AD [2]. Human proof-of-concept studies then showed nearly 2-fold greater uptake of [^11^C]PiB in AD patients relative to controls in areas of brain known to contain amyloid in AD (frontal cortex, p<0.0001), while retention was equivalent for both groups in areas known to be relatively unaffected by amyloid deposition (subcortical white matter, pons, cerebellum; p>0.2) [3]. These semi-quantitative studies were followed by fully quantitative arterial-based kinetic modeling PET studies that supported the validity of simplified (non-arterial) [^11^C]PiB PET retention outcomes that exhibited good test-retest reliability (5-10%) needed for improved study feasibility for clinical application on cross-sectional and longitudinal bases [4] and for large collaborative multi-site studies, such as the Alzheimer’s disease neuroimaging initiative (ADNI).

Early translational findings include cross-sectional evidence of amyloid deposition (i.e., [^11^C]PiB retention) in 20-30% of cognitively normal elderly controls, variable retention in subjects with mild cognitive impairment (MCI) that ranged from negligible to AD-like levels and additional findings in those at risk for the development of AD [[5], for review]. Emerging longitudinal results indicate small significant increases in [^11^C]PiB retention in AD and MCI groups, and in controls who had high baseline retention [6]. Efforts are ongoing to establish relationships between in vivo imaging measures and post-mortem measures of Aβ deposition in those scanned with [^11^C]PiB PET imaging before death. Anti-amyloid therapies have shown promise based on [^11^C]PiB PET imaging results in early treatment trials. Lastly, the development of ^18^F-labeled Aβ imaging agents (several-fold longer radioactive half-life than ^11^C) has allowed greater distribution and translational capability, with one such agent recently being approved by the U.S. Food and Drug Administration for use in the rejection of AD diagnosis in patients under evaluation for AD. A decade of translation has further clarified the need for sensitive early detection of AD pathophysiology in order to identify those who might benefit most from future therapeutic intervention.
